# Ursolic Acid Ameliorates Spinal Cord Injury in Mice by Regulating Gut Microbiota and Metabolic Changes

**DOI:** 10.3389/fncel.2022.872935

**Published:** 2022-05-04

**Authors:** Zi-Jie Rong, Hong-Hua Cai, Hao Wang, Gui-Hua Liu, Zhi-Wen Zhang, Min Chen, Yu-Liang Huang

**Affiliations:** ^1^Department of Spine Surgery, Huizhou Municipal Central Hospital, Huizhou, China; ^2^Orthopaedic Institute, Huizhou Municipal Central Hospital, Huizhou, China; ^3^Department of Orthopaedics, Huizhou Municipal Central Hospital, Huizhou, China

**Keywords:** ursolic acid, spinal cord injury, gut microbiota, inflammation, metabolic changes

## Abstract

**Background**: Spinal cord injury (SCI) damages the autonomic nervous system and affects the homeostasis of gut microbiota. Ursolic acid (UA) is a candidate drug for treating nervous system injury due to its neuroprotective and antioxidant functions. The purpose of our study was to investigate the role of UA on SCI and its mechanism.

**Methods**: UA was administered to SCI mice and the solvent corn oil was used as control. The weight of the mice was recorded daily. Mice feces were collected 21 days after surgery for 16S rRNA-amplicon sequencing and untargeted metabolomics analysis. The expressions of NF-κB, IL-1β, and TNF-α in the spinal cord and colon tissues of mice were detected by Western blot and Enzyme-linked immunosorbent assay, respectively. Immunohistochemistry was used to analyze the expression of NeuN, NF-200, and synapsin in the spinal cord tissues.

**Results**: UA treatment increased body weight and soleus muscle weight of SCI mice. UA treatment inhibited inflammatory response and protected neuronal activity in SCI mice. UA improved the relative abundance of Muribaculaceae, Lachnospiraceae_NK4A136_group, and Alloprevotell genus in the gut tract of SCI mice. SCI destroyed the Glutamine_and_D-glutamate_metabolism, Nitrogen_metabolism, Aminoacyl-tRNA_biosynthesis, and Taurine_and_hypotaurine_metabolism in the gut of mice, which might be alleviated by UA.

**Conclusions**: UA treatment could inhibit SCI progression by improving the gut environment and metabolic changes, promoting synaptic regeneration and anti-inflammatory effects.

## Introduction

Spinal cord injury (SCI) is a devastating neurological and pathological condition that causes major motor, sensory, and autonomic nerve dysfunction. Its pathophysiology includes both acute and chronic phases and contains a series of destructive events, such as ischemia, inflammatory events, and motor dysfunction (Anjum et al., 2020). SCI damages the autonomic nervous system, weakening its ability to coordinate the functions of organs throughout the body (Kigerl et al., 2018). In addition, SCI decreased the relative abundance of some beneficial symbiotic bacteria (*Lactobacillus joerii*) in the gut microbiota of mice and increased the relative abundance of some potential pathogenic bacteria (Bacteroides multiform; Du et al., 2021). These studies have proved that SCI is associated with imbalances in the gut and neuropathological states and affects the gut homeostasis of gut microbiota. Therefore, targeting neuroinflammation and gut microbiota may be a strategy for treating SCI.

Ursolic acid (UA, 3B-hydroxy-12-urc-12-en-28-oic acid) is a plant-derived pentacyclic triterpenoid known for its neuroprotective, antioxidant, and anti-inflammatory activities, and maybe a candidate for the treatment of nerve damage (Bhat et al., 2016; Sahu et al., 2018). UA could promote central nervous system (CNS) myelin regeneration and nerve repair in experimental allergic encephalomyelitis mice with complete demyelination and axon injury after modeling (Scott et al., 2002). UA also promoted myelin regeneration and oligodendrocyte maturation in the cuprizone-induced demyelinating mouse model and hippocampal organotypic brain slice model (Zhang et al., 2020). UA treatment could improve subcutaneous mTOR expression in mice with severe SCI lesions, and improve the defects of body and subinjury muscle mass caused by SCI, which may be a potential therapeutic strategy to improve SCI muscle-specific pathological consequences (Kunkel et al., 2011; Bigford et al., 2018). In general, UA could alleviate SCI-related inflammation and neurological function. However, its specific mechanism of action is still unclear.

Bidirectional communication between gut microbiota and the CNS (microbiome-gut-brain axis) is involved in brain function regulation, neural development, and aging (Cryan et al., 2019). Microbiota can communicate with the brain through neuroactive metabolites (such as neurotransmitters), microbiota products (such as short-chain fatty acids), nerves (gut nerves, vagus nerves, etc.), HPA axis, endocrine (gut hormones), immunity (immune cells and cytokines) and other pathways (Wang and Wang, 2016). Disturbances of the microbiome-gut-brain axis may be a predisposition factor for neurological disorders such as multiple sclerosis, autism, epilepsy, and Huntington’s disease (Cryan et al., 2020). It is reported that regulating gut microbiota may reduce inflammation in the CNS (Colpitts and Kasper, 2017). SCI mice receiving fecal microbiota transplantation (FMT) from healthy mice were shown to have improved axonal regeneration, weight gain, metabolic profile, gut barrier integrity, and gastrogut transport. FMT may reduce nerve and gut inflammation in SCI mice by down-regulating IL-1β/NF-κB pathway and NF-κB pathway (Jing et al., 2021).

In summary, SCI is accompanied by pathological changes in the gut and nervous systems, and drugs targeting the gut environment may alleviate SCI. UA has neuroprotective and anti-inflammatory activities, but its specific mechanism of action on spinal cord injury remains unknown. Therefore, this study intends to construct an animal model of SCI and treat it with UA, to explore whether UA could repair nerve injury and treat SCI by regulating gut microbiota.

## Materials and Methods

### Animal Models

Forty adult female C57BL/6N (18–22 g) mice were purchased from Hunan SJA Laboratory Animal Co., Ltd. Mice were kept under standard conditions (Temperature, 22 ± 2°C; Humidity, 55% ± 10%) with light-dark (12:12) cycle. The mice had free access to food and water. Forty mice were randomly divided into four groups (10 mice/group): sham, SCI, SCI+CO, and SCI+UA group. Mice were anesthetized with 2% isoflurane. After anesthesia, the laminectomy was performed to expose the T10 spinal cord and a weight (weighing 5 g) was used to fall onto the spinal cord at a height of 6.5 cm to cause injury. Mice in the sham group were exposed only to the spinal cord without contusion. The UA working solution was obtained by dissolving 20 mg UA (#77-52-1, Merck, USA) in 0.8 ml corn oil (CO). After surgery, mice in the SCI+UA group were intragastric with UA working solution (200 mg/Kg) and SCI+CO group were intragastric with CO, once a day. The mice were weighed daily. On day 21 after surgery, fresh feces samples from each mouse were collected into sterile tubes for microbiome and untargeted metabolomics sequencing. Then the mice were sacrificed and the wet weight of the soleus muscle was weighed, and the tissues of the spinal cord and colon were collected for further analysis. This study was approved by the Ethics Committee of Huizhou Municipal Central Hospital (No. kyll20211916).

### Western Blot (WB)

Radio-immunoprecipitation assay (RIPA) lysate (P0013B, Beyotime, China) was used to extract total protein from mouse spinal cord tissue. SDS-PAGE was used to isolate proteins. The target protein was transferred to nitrocellulose membrane and sealed with 5% skim milk powder for 90 min. Then, the membranes were incubated with primary antibodies NF-κB (10745-1-AP, 1:2,000, proteintech, USA), IL-1β (ab254360, 1:1,000, abcam, UK), TNF-α (17590-1-AP, 1:1,000, ProteinTech, USA), and β-actin (66009-1-Ig, 1:5,000, ProteinTech, USA) and secondary antibodies HRP anti-mouse (AWS0001a, 1:5,000, Abiowell, China) and HRP anti-rabbit (AWS0002a, 1:5,000, Abiowell, China) at room temperature for 90 min. Finally, the bands were visualized using SuperECL Plus (K-12045-D50, Advansta, USA) and photographed using Chemiscope6100 (CLiNX, China). β-actin was used as an internal reference.

### Enzyme-Linked Immunosorbent Assay (ELISA)

The concentrations of NF-κB, IL-1β, and TNF-α in mouse colon tissue were determined using ELISA KIT. Mouse IL-1β ELISA KIT (CSB-E08054m) and Mouse TNF-α ELISA KIT (CSB-E04741m) were purchased from CUSABIO (Wuhan, China). Mouse NF-κB ELISA KIT (ml058779) was obtained from Enzyme-linked Biotechnology Co., Ltd (Shanghai, China). All steps were carried out in strict accordance with the instructions. The absorbance of each well at 450 nm was measured by a microplate reader (MB-530, HEALES, China) within 5 min after the termination of the reaction.

### Immunohistochemistry (IHC)

The spinal cord tissue of mice was embedded in paraffin and made into 2 μm thick paraffin sections. The sections were baked in the microwave at 60°C for 12 h. Next, the sections were dewaxed and rehydrated using xylene and ethanol. The sections were immersed in 0.01 M citrate buffer (pH 6.0) and boiled for 23 min to repair the antigens. Sections were incubated with 1% periodate acid at room temperature for 10 min to inactivate endogenous enzymes. Sections were incubated with primary antibodies NeuN (26975-1-AP, 1:100, ProteinTech, USA), NF-200 (60331-1-Ig, 1:100, ProteinTech, USA), and synapsin (20258-1-AP, 1:100, ProteinTech, USA) at 4°C overnight. On the 2nd day, the sections were incubated with secondary antibody at 37°C for 30 min. DAB working solution (50–100 μl) was used for color rendering. The sections were re-stained with hematoxylin for 10 min. After incubation with ethanol and xylene solution, the sections were sealed with neutral gum. A light microscope (BA410T, Motic, China) was used to observe the tissue and images were analyzed with image-pro-plus software.

### 16S rRNA-Amplicon Sequencing

QIAamp^®^ Fast DNA Stool Mini Kit (Qiagen) was used to extract microbial genomic DNA from 200 mg feces samples. Agilent 4200 TapeStation (Agilent Technologies) kit was used to test the quality of DNA. Then Nextera XT DNA Sample Prep Kit (Illumina) was used to generate sequencing libraries. Agilent 4200 TapeStation was used to confirm data quality. The whole-genome sequencing was performed on Illumina NovaSeq 6000 platform. After obtaining the original data for quality control, the species composition in the samples was analyzed by comparing it with the species database.

### LC-MS Analysis and Annotation

Untargeted metabolomics was used to analyze the metabolome in mouse feces. One-hundred microliter feces was mixed with 300 μl methanol and 20 μl internal standard. Samples were extracted with ultrasound for 5 min in an ice bath and then stood at −20°C for 2 h. After centrifuging at 13,000 rpm at 4°C for 15 min, 200 μl supernatant was taken in a 2 ml vial for LC-MS analysis. The instrument platform for LC-MS analysis consisted of Agilent 1290 ULTRA performance liquid chromatography in tandem with Thermo Fisher Scientific Q Exactive Orbitrap High-resolution mass spectrometer. The chromatographic column was UPLC HSS T3 (1.7 μm 2.1 × 100 mm, Waters). Compound Discover (Version 2.0, Thermo) and OSI-SMMS (Version 1.0, Dalian Dasuo Information Technology Co., LTD.) were used in conjunction with mzCloud database and self-built database for substance identification.

### Statistical Analysis

Statistical analysis and data graphs were generated in GraphPad Prism 8.0.1 (GraphPad Software, La Jolla). Data were expressed as mean ± standard deviation. For normally distributed variables, Student’s *T*-test was used to compare the differences between the two groups. Differences among multiple groups were evaluated by one-way ANOVA followed by a Tukey multiple comparisons posttest. Qiime2 (QIIME2-2020.2) and R software (4.0.2) were used for sequence data analysis. Qiime 2 software was used to calculate the Alpha diversity index (including Chao1, ACE, Shannon, Simpson evenness index, etc.) of all samples. Kruskal Wallis test (between multiple groups) and Wilcoxon test (between two groups) were used to plot the box plot (R phyloseq package) of α diversity between groups. R software was used to draw a histogram of relative species abundance (R GGplot2 package), heatmap of genus abundance (R reshape2/GGplot2 package), Anosim analysis, and differential analysis of species abundance based on Wald test (R DESeq2 package). In addition, the Veen figure^1^ and Lefse analysis^2^ were performed with the help of the net Page analysis tools. Pearson correlation analysis was used to analyze the correlation between variables. *P* < 0.05 was considered statistically significant.

## Results

### UA Improved the Motor Function of SCI Mice

**Figure 1:**
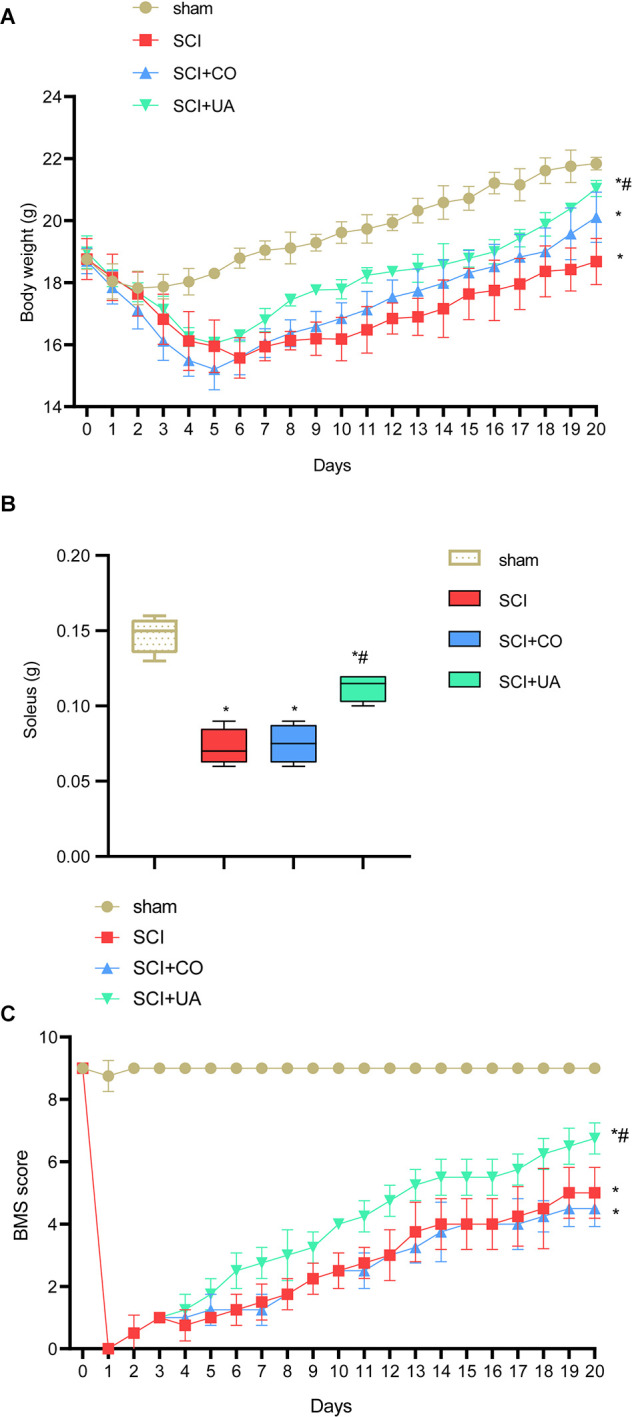
UA improved the motor function of SCI mice. **(A)** The bodyweight of mice at different times. **(B)** The weight of mice soleus. **(C)** BMS score was applied to monitor the motor function recovery. Data were expressed as mean ± standard deviation; each experiment was repeated at least three times; *n* = 3; differences among multiple groups were evaluated by one-way ANOVA followed by a Tukey multiple comparisons post-test. *P < 0.05 vs. the sham group; ^#^*P* < 0.05 vs. the SCI+CO group.

Motor neurons located in the spinal cord innervate the leg muscles and regulate the neural circuits involved in motor control (Knikou and Murray, 2019). To study the effect of UA treatment on the locomotor ability of SCI mice, the weight of the body and soleus muscle of mice were measured. As shown in Figures 1A,B, compared with the sham group, the body weight and soleus muscle weight of mice in the SCI group were reduced. After CO treatment, the bodyweight of mice increased, but the muscle mass of the soleus did not change significantly. UA treatment significantly increased body weight and soleus muscle weight in mice. The soleus muscle plays a very important role in animal standing, walking, running, and jumping. BMS scores could monitor motor function recovery. As shown in Figure 1C, the BMS score of the SCI group was significantly lower than that of the sham group. With the extension of time, the BMS score showed an upward trend, but it was always lower than the sham group. However, there was no significant change in BMS score of mice after CO treatment. UA significantly improved the BMS score of mice. These results suggested that UA treatment could restore body weight, exercise ability in SCI mice to some extent.

### UA Inhibited Inflammation and Promoted Neuronal Survival and Synaptic Regeneration

**Figure 2:**
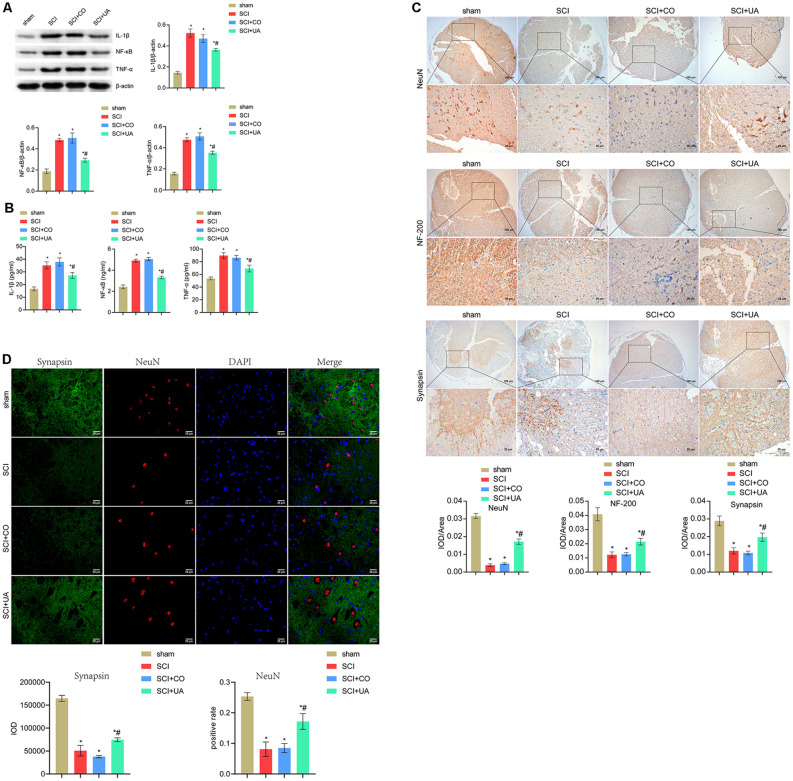
UA inhibited inflammation and promoted neuronal survival and synaptic regeneration. **(A)** WB was performed to detect the protein expression of IL-1β, NF-κB, and TNF-α. **(B)** The concentration of IL-1β, NF-κB, and TNF-α was measured by ELISA. **(C)** IHC was conducted to analyze the expression of NeuN, NF-200, and synapsin. **(D)** Immunofluorescence double staining for NeuN and synapsin. Data were expressed as mean ± standard deviation; each experiment was repeated at least three times; *n* = 3; differences among multiple groups were evaluated by one-way ANOVA followed by a Tukey multiple comparisons posttest. *P < 0.05 vs. the sham group; ^#^*P* < 0.05 vs. the SCI+CO group; Magnification = 400 times; Scale bar = 25 μm.

Next, we investigated the effects of UA on inflammation in the gut tract and spinal cord, as well as neuronal survival and synaptic regeneration in mice. Pro-inflammatory cytokines such as TNF-α and IL-1β play an important role in the regulation of immune response (Jablonska et al., 1997). Compared with the sham group, the expression levels of IL-1β, NF-κB, and TNF-α in the spinal cord and gut tract of the SCI group were significantly increased. After UA treatment, the IL-1β, NF-κB, and TNF-α expressions were decreased (Figures 2A,B). This showed that UA could inhibit the inflammatory response in SCI mice. As shown in Figure 2C, UA treatment reversed the decreased expression of NeuN, NF-200, and synapsin in SCI mice. Then we performed double immunofluorescence staining for NeuN and synapsin to examine the contact between neurons and synapses. As shown in Figure 2D, compared with the sham group, the positive rate of NeuN in the SCI group decreased, and the IOD value of synapsin also decreased. There was no significant difference in the positive rate of NeuN and the IOD value of synapsin in mice treated with CO. UA significantly increased the positive rate of NeuN and the IOD value of synapsin. These results showed that UA could protect the activity of neurons and promote synaptic regeneration in SCI mice.

### UA Improved Gut Microbiota Diversity in SCI Mice

**Figure 3:**
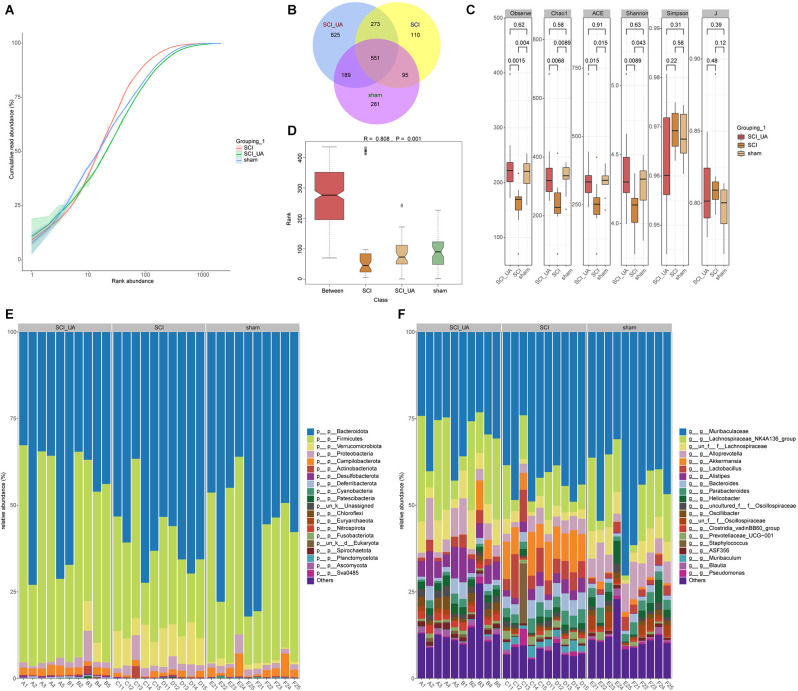
UA improved gut microbiota diversity in SCI mice. **(A)** The cumulative read abundance of gut microbiota in mice. **(B)** Venn diagrams showed the number of gut microbes in different groups. **(C)** Alpha diversity was calculated. **(D)** Anosim was used to analyze the significance of differences between groups. The heat map showed the 20 species with the highest relative abundance at **(E)** phylum and **(F)** genus levels.

In order to study the effects of UA on gut microbiota in mice, 16S rRNA-amplicon sequencing was performed on the feces of mice. Figure 3A showed the cumulative read abundance of gut microbiota. As seen from Figure 3A, the species abundance in the sham group was close to the SCI+UA group, while the species abundance in the SCI group was low. There were 551 species in common among the three groups and 281 species in the sham group, 110 species in the SCI group and 625 species in the SCI+UA group (Figure 3B). Figure 3C showed that Observe, Chao1, ACE, and Shannon indexes in the SCI group were significantly smaller than those in the sham group. After UA treatment, these indexes increased. The change in the J index was the opposite. Interestingly, there was no significant difference in the Simpson index between the SCI and sham groups. In other words, the number of gut microbiota in the SCI mice increased after UA treatment. Anosim analysis results were *R* = 0.808, *P* = 0.001, indicating that the differences among groups were significantly greater than inter-group differences, indicating significant grouping (Figure 3D). At the phylum level, Bacteroidota, Firmicutes, and Verrucomicarobiota were the most abundant bacteria in all samples (Figure 3E). In addition, Muribaculaceae, Lachnospiraceae_NK4A136_group, and Lachnospiraceae occupy a dominant position among all samples at the genus level (Figure 3F). These results indicated that SCI mice had reduced gut microbial diversity, which can be ameliorated by UA treatment.

### UA Improved the Dominant Gut Microbiota of SCI Mice

**Figure 4:**
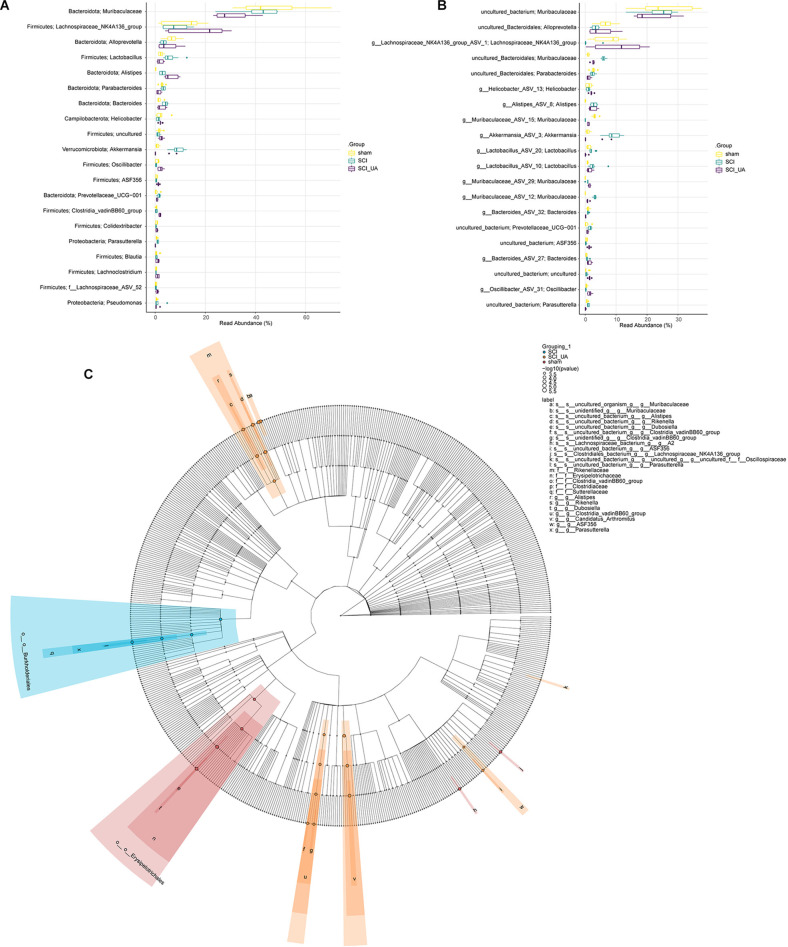
UA improved the dominant gut microbiota of SCI mice. Boxplots were used to compare the 20 species with the highest relative abundance at **(A)** Phylum_Genus and **(B)** Species_Genus levels in each group. **(C)** LEfSE analysis was performed to seek the difference among groups.

Then, we sorted the species in order of relative abundance from large to small, and selected TOP 20 to draw a boxplot. Figures 4A,B showed the differences in the abundance of the main species at Phylum_Genus and Species_Genus levels. Figure 4A showed that compared with the sham group, the relative abundance of Firmicutes; Lachnospiraceae_NK4A136_group and Bacteroidota; Alloprevotella in the SCI group decreased significantly, while Bacteroidota; Muribaculaceae has no obvious change. After UA treatment, the relative abundance of Firmicutes; Lachnospiraceae_NK4A136_group, and Bacteroidota; Alloprevotella increased significantly. The relative abundance of Bacteroidota; Muribaculaceae decreased in the meantime. In addition, At the Species_Genus level, UA treatment increased the relative abundance of uncultured_Bacteroidota; Alloprevotella, g_Lachnospiraceae_NK4A136_group_ASV_1; Lachnospiraceae_NK4A136_group, and g_Muribaculaceae _ASV_15; Muribaculaceae. Interestingly, the relative abundance of Muribaculaceae significantly decreased in the SCI_UA group (Figure 4B). Lefse analysis further confirmed that there were significant differences between Muribaculaceae and Lachnospiraceae_NK4A136_group (Figure 4C). These results reflected that Muribaculaceae, Lachnospiraceae_NK4A136_group, and Alloprevotella might play an important role in the development and onset of SCI.

### UA Regulated Gut Metabolism in SCI Mice

**Figure 5:**
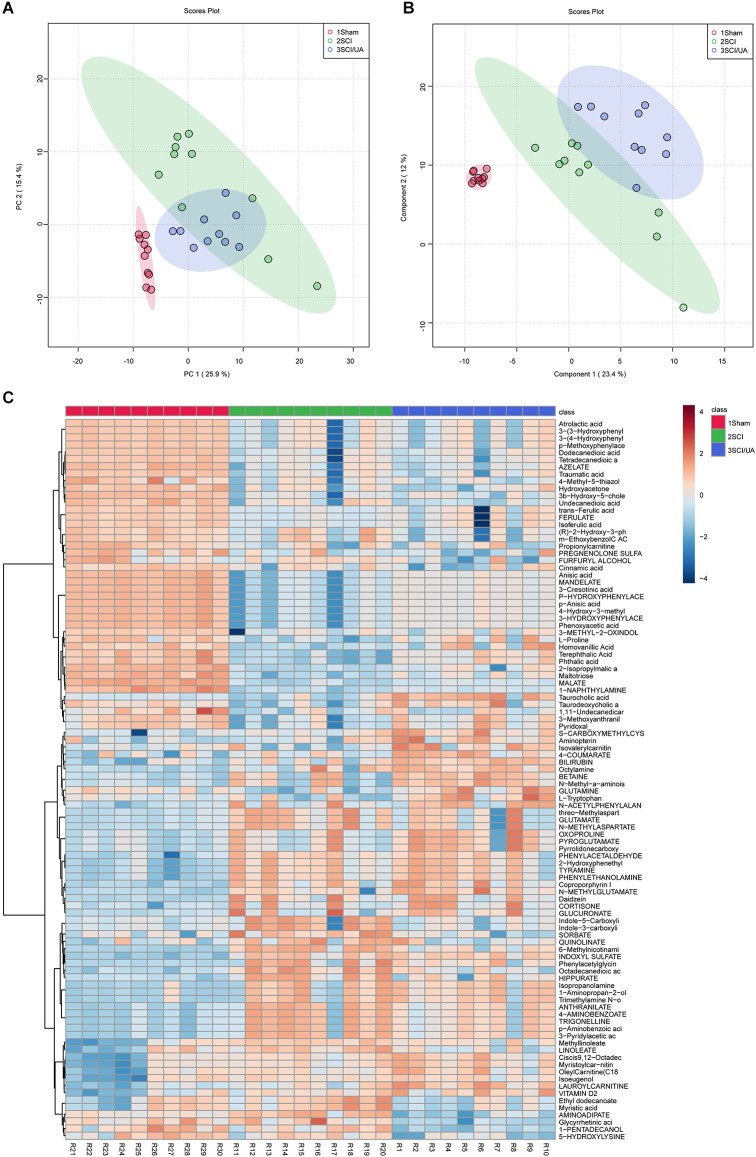
UA regulated gut metabolism in SCI mice. **(A)** PCA and **(B)** PLS-DA was performed for dimension reduction analysis of metabolites in mice. **(C)** The heatmap showed the top 100 metabolites.

In order to study the effects of UA on gut metabolism, we used an untargeted metabolomics method to screen endogenous small molecules with a relative molecular weight less than 1,000 in feces. After the metabolites were characterized, Principal Component Analysis (PCA, Figure 5A) and Partial Least Squares Discriminant Analysis (PLS-DA, Figure 5B) were performed. PCA is an unsupervised multidimensional statistical analysis method, which can reflect the overall metabolic difference between each group and the variation degree between samples within the group. PCA showed that the principal component PC1 index was 25.9% and the PC2 index was 15.4%. PLS-DA is a supervised discriminant analysis method. The PLS-DA index of differential factor variables was 23.4%. Heatmap showed the top 100 metabolites obtained by untargeted screening in feces. Both the Sham group and SCI group were enriched with dominant metabolites and were significantly different, while UA treatment could narrow the difference to a certain extent (Figure 5C). The top three dominant metabolites were atrolactic acid, 3-(3-hydroxyphenyl) propionate acid, and 3-(4-hydroxyphenyl) propionic acid. These results suggested that SCI mice have altered gut metabolome that can be reversed by UA.

### Intestinal Metabolic Function Changes in SCI Mice

**Figure 6:**
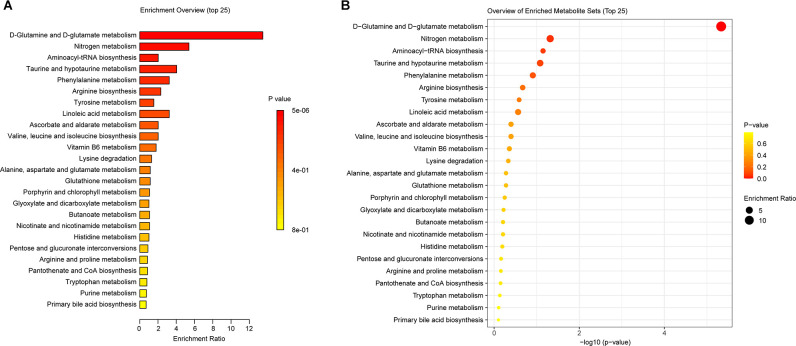
KEGG was used to analyze the gut metabolic function in mice. **(A)** The enrichment ratio of KEGG pathways. **(B)** Bubble diagram showed the *p*-value of these enrichments.

To further investigate the functional changes caused by intestinal metabolite changes in SCI mice. We used the KEGG PATHWAY Database^3^ functional annotation platform function prediction signal path analyses. Figures 6A,B showed that in the three groups of mice, the metabolic pathway D-glutamine_and_D-glutamate_metabolism, Nitrogen_metabolism, aminoacyl-Trna_biosynthesis, and Taurine_and_hypotaurine_metabolism changed significantly. The main differential metabolites in these metabolic pathways were C0064 (L-Glutamine), C00025 (L-Glutamate), C0073 (L-Methionine), C00183 (L-Valine), C00078 (L-Tryptophan), C00148 (L-Proline), C02237 (5-Oxo-D-proline), C00245 (Taurine), and C05122 (Taurocholate). In other words, UA may alleviate SCI by regulating the metabolic level of mice.

### UA Affects Gut Microbiota and Metabolism in SCI Mice, Thus Regulating Inflammation and Neural Function

**Figure 7:**
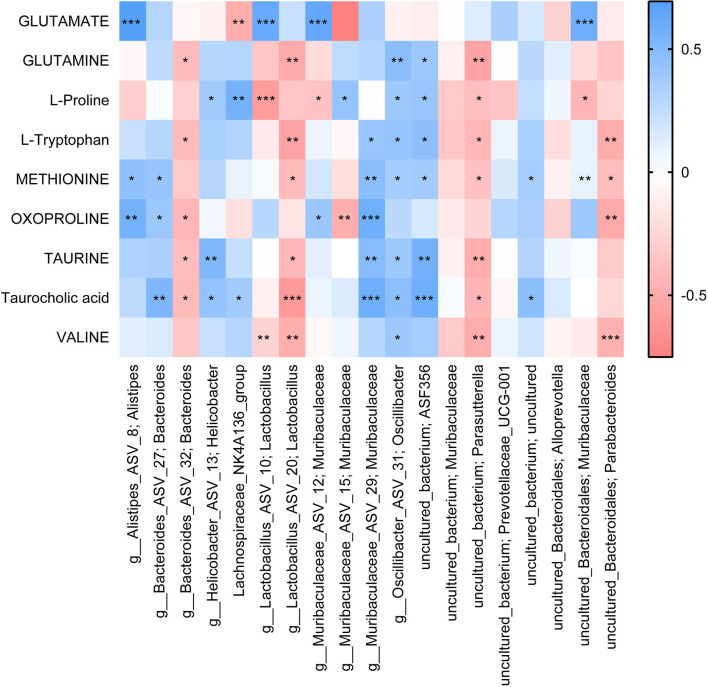
Correlation analysis of differential metabolites with the top 20 gut species. Each block represents a correlation, and the color of the block represents the strength of the correlation. A high absolute value of R-value indicates a strong correlation. *P < 0.05. **P < 0.01. ***P < 0.001.

**Figure 8:**
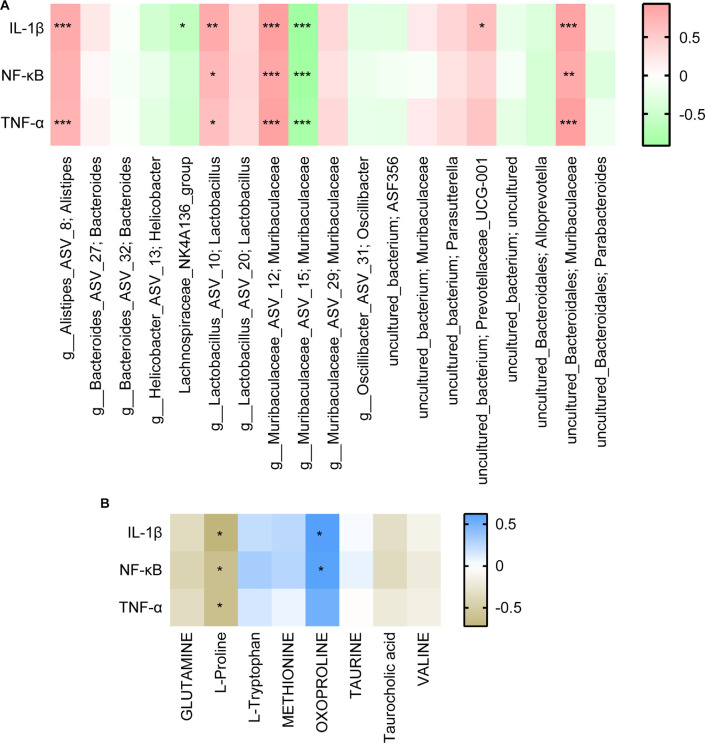
Correlation analysis of inflammatory factors with **(A)** the top 20 gut species and **(B)** differential metabolites. Each block represents a correlation, and the color of the block represents the strength of the correlation. A high absolute value of R value indicates a strong correlation. *P < 0.05. **P < 0.01. ***P < 0.001.

Finally, we analyzed the correlation between dominant microbes and differential metabolites in the gut tract of mice. According to Figure 7, The relative abundance of Lachnospiraceae_NK4A136 _group, g_Muribaculaceae_ASV_15; Muribaculaceae, and g_Muribaculaceae_ASV_12; Muribaculaceae was significantly correlated with GLUTAMATE concentration. Figure 8A showed that the relative abundance of g_Muribaculaceae_ASV_12; Muribaculacea, g_Muribaculaceae_ASV_15; Muribaculaceae, and uncultured_Bacteroidales; Muribaculaceae was significantly correlated with the expression of IL-1β, NF-κB, and TNF-α. Besides that, the concentration of differential metabolite L-Proline was also significantly correlated with the expression of inflammatory factors (Figure 8B). These results indicated that UA might affect metabolic function by regulating gut microbiota, thereby affecting inflammation and neuronal activity in mice, and ultimately alleviating SCI in mice.

## Discussion

There is a growing study show that SCI is a two-step process. Primary SCI refers to the initial mechanical injury caused by local deformation of the spine (Ambrozaitis et al., 2006). The primary injury is followed by a progressive cascade of secondary injuries characterized by apoptosis of neurons and glial cells (especially oligodendrocytes), axonal retraction, glial scarring, and recruitment of inflammatory cells (Sandrow-Feinberg and Houlé, 2015). The neuroinflammatory response is an important contributing factor to SCI-induced secondary injury (Chen et al., 2018). In addition, neuronal dysfunction is a hallmark of spinal cord injury (SCI), and treatment efforts usually focus on axonal regeneration in the CNS (Orr and Gensel, 2018). Synaptic proteins are involved in the synaptic formation and plasticity of mature synapses and play an important role in maintaining brain physiology. NeuN is a biomarker of arcuate neurons and has neuroprotective effects (Kim et al., 2009). NF-200 could promote neurite outgrowth, while synapsin is associated with synaptic vesicles (Shen et al., 2017). In our study, we found that UA treatment reversed the decreased expression of NeuN, NF-200, and synapsin in SCI mice. Our study demonstrated that UA treatment inhibited the inflammatory response of SCI mice and enhanced the motor ability and neuronal activity of SCI mice. It is reported that UA could reduce inflammation by inhibiting the NF-κB signaling in cancer cells (Seo et al., 2018). Furthermore, UA could induce nerve regeneration after sciatic nerve injury (Liu et al., 2013). In other words, we confirmed the therapeutic effect of UA on SCI mice.

Then, to analyze the mechanism of UA, we detected the gut microbiota composition of each group of mice. We found that Muribaculaceae and Lachnospiraceae_NK4A136_group were dominant among all mouse gut microbiota at genus. Other than that, UA treatment increased the Lachnospiraceae_NK4A136_group and Alloprevotella, and decreased the Muribaculaceae. Interestingly enough, the relative abundance of g_Muribaculaceae_ASV_15; Muribaculaceae, and g_Muribaculaceae_ASV_12; Muribaculaceae showed a completely opposite change. Short-chain fatty acids have been shown to maintain stem cell populations and inhibit gut inflammation by inhibiting histone deacetylase (Meng et al., 2020). Lachnospiraceae_NK4A136_groups produce short-chain fatty acids, which are thought to be anti-inflammatory factors and inhibit the inflammatory response in DSS-induced colitis (Dou et al., 2020). Previous studies have shown that Lachnospiraceae_NK4A136_group is decreased in obese subjects, and its changes are relevant with spermidine-induced upturn of gut barrier function (Li et al., 2019). Similarly, resveratrol reversed the dysregulation of Alloprevotella, inhibiting inflammation and preventing diabetic nephropathy in mice (Cai et al., 2020). Temozolomide treatment in a glial mouse model increases the Muribaculaceae family, which in turn regulates inflammatory responses in the gut (Patrizz et al., 2020). Our correlation analysis showed that Lachnospiraceae_NK4A136_group and Alloprevotella were negatively correlated with inflammatory factors. Meanwhile, inflammatory factors were significantly positively correlated with most Muribaculaceae species. It suggested that UA-mediated changes in gut microbiota may play an essential part in the mechanism of UA.

In metabolomics, we successfully screened the top 100 differential metabolites in the serum of three groups of mice. KEGG was used to enrich the differential metabolites and four major differential metabolic pathways were screened out: D-Glutamine_and_D-glutamate_metabolism, Nitrogen_metabolism, Aminoacyl-tRNA_biosynthesis, and Taurine_and_hypotaurine_metabolism. Glutamine is released by astrocytes, which is a precursor of neurotransmitters (Bak et al., 2006). Glutamate is the main excitatory neurotransmitter in the CNS of mammals (Zhou and Danbolt, 2014). Nitrogen_metabolism is thought related to neuron-astrocytes (Yudkoff et al., 1992). Furthermore, genetic variation of Aminoacyl-tRNA synthetases leads to neurodegenerative peripheral neuropathy and damage to the movement and sensory function of the distal limbs (Kuo and Antonellis, 2020). In addition, Taurine and Hypotaurine are cysteamine metabolites and have shown strong neuroprotective effects against mutant Huntington toxin (Arbez et al., 2019). These metabolic variations all point to neuroprotection and regeneration. In other words, our study demonstrated that UA-mediated metabolic changes may be the pathway of neural repair and regeneration. In addition, we found that gut microbiota is correlated with metabolites. These correlated changes in gut microbiota and metabolites suggest that gut microbiota may be involved in SCI through interactions with various metabolites. Meanwhile, these specific metabolites may be influenced or produced by the corresponding gut microbiota.

In general, UA therapy countered SCI progression by improving the gut environment and metabolic changes, and these changes might be an important mechanism by which UA mediates its beneficial synaptic regeneration and anti-inflammatory effects. These findings provide supporting evidence for the use of UA in the treatment of SCI. However, our study has many limitations. In future sequencing experiments, more samples should be selected. Furthermore, in human studies, the specific effects of UA on gut microbiota and the development of SCI remain unclear. More clinical trials are needed to confirm these and other possible effects and mechanisms.

## Data Availability Statement

The data presented in the study are deposited in the NCBI BioProject repository, accession number PRJNA811708.

## Ethics Statement

The animal study was reviewed and approved by the Ethics Committee of Huizhou Municipal Central Hospital (No. kyll20211916).

## Author Contributions

MC and Y-LH contributed to the study conception and design. Material preparation, data collection and analysis were performed by Z-JR, H-HC, HW, G-HL, and Z-WZ. The first draft of the manuscript was written by Z-JR. All authors agree to be accountable for the content of the work. All authors contributed to the article and approved the submitted version.

## Conflict of Interest

The authors declare that the research was conducted in the absence of any commercial or financial relationships that could be construed as a potential conflict of interest.

## Publisher’s Note

All claims expressed in this article are solely those of the authors and do not necessarily represent those of their affiliated organizations, or those of the publisher, the editors and the reviewers. Any product that may be evaluated in this article, or claim that may be made by its manufacturer, is not guaranteed or endorsed by the publisher.

## Footnotes

^1^ http://www.bioinformatics.com.cn/static/others/jvenn/example.html

^2^ https://github.com/SegataLab/lefse

^3^ https://www.kegg.jp/kegg/pathway.html
